# MRI Dark–Light–Dark Sign and Node Reporting Score: An Integrated Model for Predicting Lymph Node Metastasis in Rectal Cancer

**DOI:** 10.3390/jcm15135146

**Published:** 2026-07-01

**Authors:** Cenk Parlatan, Şeyda Gökçe Turunç, Okan Dilek, Zeynel Abidin Taş, Görkem Özdemir, Timuçin Çil

**Affiliations:** 1 Department of Radiology, Adana Dr. Turgut Noyan Application and Research Center, Baskent University, Adana 01250, Turkey; 2 Department of Radiology, Adana City Training and Research Hospital, University of Health Sciences, Adana 01230, Turkey; 3 Department of Pathology, Adana City Training and Research Hospital, University of Health Sciences, Adana 01230, Turkey; 4 Department of Gastroenterological Surgery, Adana City Training and Research Hospital, University of Health Sciences, Adana 01230, Turkey; 5 Department of Medical Oncology, Adana City Training and Research Hospital, University of Health Sciences, Adana 01230, Turkey

**Keywords:** rectal cancer, lymph node metastasis, node reporting and data system, dark–light–dark sign, magnetic resonance imaging

## Abstract

**Objectives:** To evaluate the diagnostic performance and incremental value of the dark-light-dark (DLD) sign combined with the Node Reporting and Data System (Node-RADS) for the preoperative prediction of lymph node metastasis in rectal cancer. **Methods:** This retrospective single-center study included 166 patients with histopathologically confirmed rectal adenocarcinoma who underwent preoperative pelvic MRI and subsequent histopathological nodal assessment. Node-RADS score and DLD status were independently assessed by three blinded radiologists, followed by consensus review. Multivariable logistic regression was used to construct a combined DLD + Node-RADS model. Model discrimination was evaluated using receiver operating characteristic analysis and DeLong’s test. Calibration, bootstrap internal validation, and decision curve analyses were performed to assess the model’s reliability and potential clinical utility. **Results:** Histopathological lymph node metastasis was observed in 122/166 patients (73.5%). Both the Node-RADS score and DLD-negative status were significantly associated with nodal metastasis. In multivariable analysis, the Node-RADS score remained independently associated with lymph node metastasis (adjusted OR, 2.054; 95% CI, 1.455–2.899; *p* < 0.001), and DLD-negative status was an independent predictor after adjustment for Node-RADS (adjusted OR, 6.635; 95% CI, 2.873–15.319; *p* < 0.001). The combined model achieved higher discriminatory performance (AUC, 0.795; 95% CI, 0.715–0.875) than Node-RADS alone (AUC, 0.740) and DLD status alone (AUC, 0.713), with significant improvements according to DeLong analysis. Calibration analysis demonstrated good agreement between predicted and observed outcomes (Brier score, 0.142; Hosmer–Lemeshow *p* = 0.101), and bootstrap internal validation suggested stable performance. Decision curve analysis showed a greater net benefit for the combined model across clinically relevant threshold probabilities. **Conclusions:** The integration of DLD status with Node-RADS showed potential incremental value for preoperative prediction of lymph node metastasis compared with either approach alone. The combined model demonstrated good calibration, stable internal performance, and potential clinical utility. This imaging-based strategy may provide complementary risk stratification, particularly in indeterminate nodal categories, although external validation is required before routine clinical implementation.

## 1. Introduction

Rectal cancer is a major cause of cancer-related morbidity and mortality worldwide [1]. Lymph node metastasis is one of the most important prognostic factors in rectal cancer and directly influences decisions regarding neoadjuvant therapy and surgical management [2,3]. Therefore, accurate preoperative prediction of nodal involvement is essential for optimal treatment planning in these patients.

Current MRI-based nodal assessment relies not only on lymph node size but also on morphological features such as border irregularity, shape, and signal heterogeneity [4,5]. However, despite these criteria, the diagnostic accuracy of MRI for nodal staging remains limited because of the substantial overlap between benign and metastatic lymph nodes [6]. Small metastatic nodes may be underestimated, whereas reactive nodes may lead to false-positive interpretations.

Structured scoring systems integrating multiple imaging features have been introduced to improve diagnostic performance. The Node Reporting and Data System (Node-RADS) provides standardized nodal risk stratification by combining lymph node size, shape, border characteristics, and internal signal features [7]. Although previous studies have demonstrated promising diagnostic performance, uncertainty remains, particularly in intermediate-risk categories, where clinical decision-making is often difficult [8,9].

Recently, Yan et al. described the “dark–light–dark” (DLD) pattern on contrast-enhanced MRI as a potential imaging biomarker associated with lymph node metastasis in rectal cancer [10]. The DLD sign is characterized by alternating intratumoral signal-intensity layers on contrast-enhanced imaging. According to the original description, preservation of the DLD sign is generally associated with less aggressive tumor behavior, whereas loss of the DLD sign may reflect stromal heterogeneity, fibrosis, desmoplastic reaction, vascular alterations, and other microenvironmental changes associated with a higher likelihood of lymph node metastasis. Initial findings suggest that DLD status may provide complementary information beyond conventional morphologic nodal assessment; however, external validation remains limited. To our knowledge, no previous study has evaluated the incremental value of integrating DLD status with the Node-RADS classification system for preoperative lymph node assessment in rectal cancer.

Therefore, this study aimed to evaluate and compare the diagnostic performance of the DLD sign and Node-RADS scoring system, individually and in combination, for predicting lymph node metastasis in rectal cancer.

## 2. Materials and Methods

This retrospective, single-center, observational study included patients with histopathologically confirmed rectal adenocarcinoma treated between January 2017 and March 2025. Institutional review board approval was obtained (19 February 2026/1096), and informed consent was waived because of the retrospective study design. This study was conducted in accordance with the Declaration of Helsinki.

### 2.1. Patient Selection

A total of 205 consecutive patients were identified through hospital and radiology archive records. The inclusion criteria were as follows: (i) histopathologically confirmed rectal adenocarcinoma before surgery, (ii) availability of pre-treatment high-resolution pelvic magnetic resonance imaging (MRI), and (iii) availability of postoperative histopathological lymph node evaluation.

**Figure 1:**
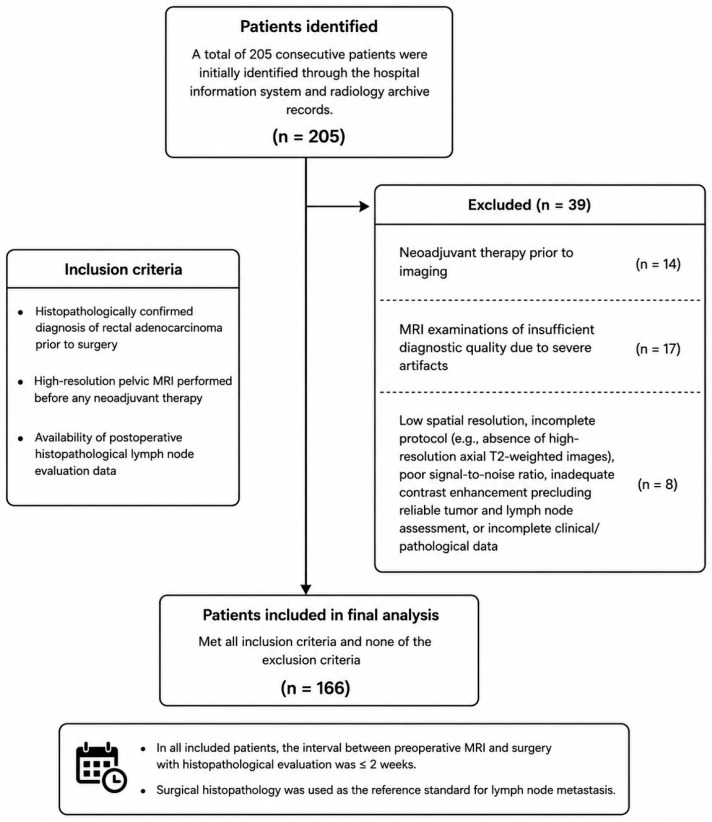
Flowchart of patient selection, exclusion criteria, and final study cohort inclusion for the analysis of lymph node metastasis in rectal cancer.

To ensure cohort homogeneity, patients who underwent neoadjuvant therapy before imaging (n = 14), patients with severe MRI artifacts compromising diagnostic quality (n = 17), and patients with low spatial resolution, incomplete imaging protocols (e.g., absence of high-resolution axial T2-weighted imaging), poor signal-to-noise ratio, inadequate contrast enhancement preventing reliable tumor or lymph node evaluation, or incomplete clinical or pathological data (n = 8) were excluded (Figure 1).

Based on these criteria, 166 patients were included in the final analysis. Demographic and clinical data were obtained from the electronic medical record system. To minimize interval-related changes in tumor characteristics and nodal status, only patients with an MRI-to-surgery interval of ≤2 weeks were included. The median interval between MRI and surgery was 9 days.

Histopathological examination of surgical specimens served as the reference standard for diagnosing lymph node metastasis. All specimens were evaluated by a gastrointestinal pathologist with 15 years of experience (Z.A.T.), who was blinded to all MRI findings, including Node-RADS categorization and DLD status. Patients with histopathologically confirmed lymph node metastasis were classified as node-positive, whereas those without metastasis were classified as node-negative. Imaging findings and pathological outcomes were compared on a per-patient basis because node-specific radiologic–pathologic matching was not available in this retrospective cohort.

### 2.2. MRI Protocol

Magnetic resonance imaging was performed using a 3-T MR scanner (Philips Ingenia; Philips Healthcare, 2017, Best, The Netherlands) equipped with a phased-array surface body coil. No changes were made to the scanner hardware, coil configuration, or imaging protocol during the study period.

For T2-weighted axial, coronal, and sagittal sequences, the matrix size was 220 × 205, the field of view (FOV) was 220 mm, the slice thickness was 3 mm, the interslice gap was 0.5 mm, and the repetition time/echo time (TR/TE) was 3299/110 ms. For pre-contrast and post-contrast T1-weighted axial and sagittal sequences, the matrix size was 312 × 224, FOV was 250 mm, slice thickness was 4 mm, interslice gap was 0.4 mm, and TR/TE was 514/8 ms.

For dynamic contrast-enhanced axial T1-weighted imaging, the matrix size was 220 × 223, FOV was 240 mm, TR/TE was 6.0/1.88 ms, dynamic scan time was 10.5 s, and k0 time was 3.0 s.

Diffusion-weighted imaging covering the entire pelvis was performed using b values of 0 and 1000 s/mm^2^. High-spatial-resolution oblique axial, sagittal, and coronal T2-weighted images without fat suppression were obtained perpendicular or parallel to the long axis of the tumor.

All patients received 0.1 mmol/kg gadobutrol (Bayer Schering Pharma, Berlin, Germany) intravenously at 2 mL/s, followed by a 15 mL saline flush at the same rate, using an automatic injector.

MRI examinations were performed using consistent institutional imaging protocols throughout the study period.

### 2.3. Image Analysis

Lymph node assessment was performed using the Node Reporting and Data System (Node-RADS) scoring system [7]. All visible mesorectal lymph nodes adjacent to the primary tumor were evaluated, and their short-axis diameters were measured. In addition to size, morphological features, including shape (oval/kidney-shaped vs. round), margin characteristics (smooth vs. irregular), internal structure (homogeneity, necrosis, or cystic change), preservation of the fatty hilum, and MRI signal characteristics were analyzed.

Based on these findings, each lymph node was assigned a Node-RADS score from 1 to 5, representing very low to very high probability of malignancy, respectively. In patients with multiple lymph nodes, the highest Node-RADS score was used for the analysis. This patient-based approach was adopted to ensure direct comparison with the histopathological reference standard, which was available only at the patient level.

Consistent with previous Node-RADS studies, scores ≥ 3 were considered positive for metastatic involvement because category 3 represents an indeterminate but suspicious group with increased sensitivity for nodal metastasis detection [7,11,12,13].

**Figure 2:**
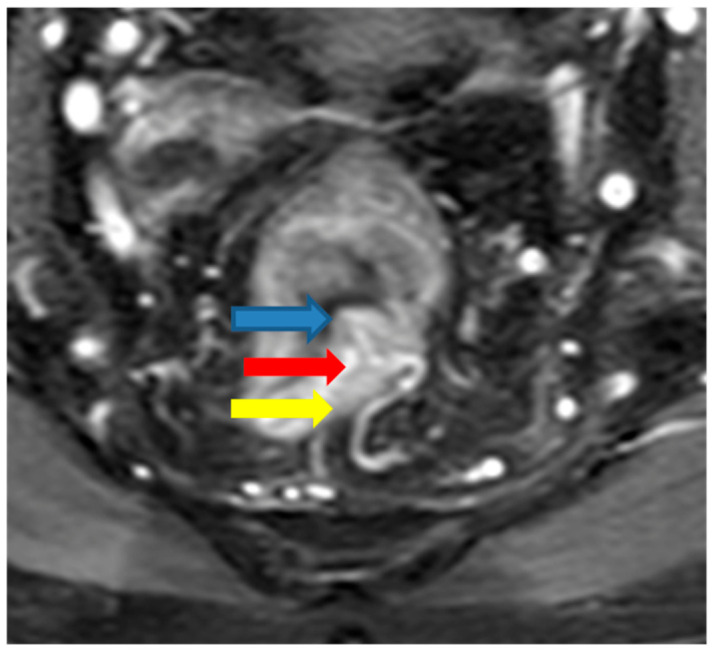
Axial contrast-enhanced T1-weighted MR image demonstrating the dark–light–dark (DLD) sign in rectal adenocarcinoma. The central hypointense tumor core (blue arrow) is surrounded by an enhancing rim (red arrow) and an outer low-signal band corresponding to the muscular layer (yellow arrow). Postoperative histopathology revealed no lymph node metastasis (node-negative).

Primary tumor evaluation was performed using the dark–light–dark (DLD) sign. On contrast-enhanced T1-weighted images, the DLD sign was defined as a trilaminar appearance consisting of a relatively hypointense tumor center, a hyperenhancing intermediate rim, and an outer low-signal layer corresponding to the muscularis propria [10]. The presence of this pattern was classified as DLD-positive (Category I), whereas its absence was classified as DLD-negative (Category II). Assessment was performed on the most representative tumor sections using multiplanar images (Figure 2).

Before image analysis, a calibration session was conducted to standardize the Node-RADS categorization and DLD sign interpretation. Representative cases not included in the study cohort were jointly reviewed by all readers to harmonize imaging criteria and reporting methodology.

Image analyses were independently performed by three radiologists with 3, 10, and 15 years of experience in rectal MRI, respectively, all blinded to clinical information, pathological results, and each other’s imaging assessments.

(Ş.G.T, C.P., and O.D.). Each reader recorded the Node-RADS score and DLD status using a standardized evaluation form.

Inter-reader agreement for Node-RADS categorization and DLD assessment was evaluated using Fleiss’ kappa (κ) statistic. Kappa values were interpreted according to the Landis and Koch criteria.

**Figure 3:**
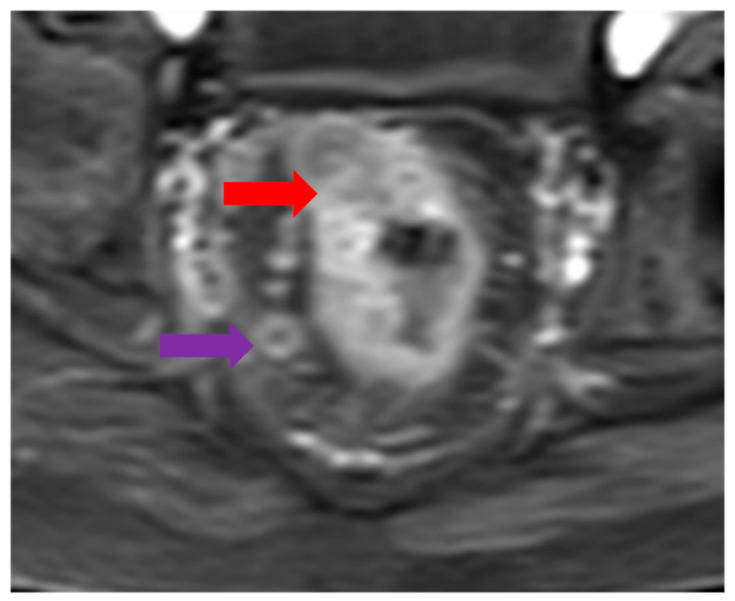
Axial contrast-enhanced T1-weighted MR image in a patient with rectal adenocarcinoma. The primary tumor (red arrow) does not demonstrate the dark–light–dark (DLD) sign. A Node-RADS 4 mesorectal lymph node is observed (purple arrow). Postoperative histopathology confirmed lymph node metastasis.

After an independent review, discrepant cases were reassessed during a structured consensus meeting involving all three radiologists. Final decisions were reached by consensus using predefined imaging criteria under standardized workstation conditions. The final Node-RADS and DLD variables used for statistical analysis were based on consensus evaluations to reduce reader dependency and improve interpretive consistency (Figure 3).

### 2.4. Statistical Analysis

All statistical analyses were performed using IBM SPSS Statistics for Windows, Version 28.0 (IBM SPSS Statistics). Continuous variables were expressed as mean ± standard deviation or median (minimum–maximum), as appropriate, whereas categorical variables were presented as frequencies and percentages.

Associations between lymph node metastasis and Node-RADS score or DLD status were evaluated using the chi-square (χ^2^) test. A two-sided *p*-value < 0.05 was considered statistically significant.

To evaluate the independent contribution of DLD status beyond the conventional Node-RADS assessment, a multivariable binary logistic regression model was constructed with histopathologically confirmed lymph node metastasis as the dependent variable. Node-RADS score was entered as an ordinal predictor (scores 1–5), and DLD status was entered as a binary predictor (DLD-positive = 0, DLD-negative = 1). The model was specified as follows:

logit(P[lymph node metastasis]) = β0 + β1 × Node-RADS score + β2 × DLD status.

Regression coefficients (β), odds ratios (ORs), 95% confidence intervals (CIs), and *p*-values were reported. The combined DLD + Node-RADS model was evaluated to investigate the complementary contribution of DLD status to the morphology-based nodal assessment. The predicted probabilities generated from the multivariable logistic regression model were subsequently used for ROC curve analysis of the combined DLD + Node-RADS model.

Univariate logistic regression analysis was initially performed to evaluate the association between individual imaging variables and lymph node metastasis. Variables demonstrating statistical significance and/or clinical relevance were entered into the multivariable logistic regression model. The results are reported as regression coefficients (β), odds ratios (ORs), 95% confidence intervals (CIs), and *p*-values. Pairwise comparisons of the AUC values between the combined model, Node-RADS alone, and DLD sign alone were performed using the DeLong method.

The diagnostic performance was evaluated using receiver operating characteristic (ROC) curve analysis, and the area under the curve (AUC) was calculated for Node-RADS, DLD status, and the combined model.

Model calibration was assessed using the Brier score, calibration intercept, calibration slope, and Hosmer–Lemeshow goodness-of-fit test. The calibration performance was evaluated using a calibration plot comparing the observed and predicted probabilities of lymph node metastasis. Internal validation of the combined DLD + Node-RADS model was performed using 1000 bootstrap resamples to assess model stability and potential optimism in diagnostic performance. Decision curve analysis was performed to evaluate the clinical utility of the combined DLD + Node-RADS model across a range of threshold probabilities. The net benefit was calculated and compared with Node-RADS alone, DLD sign alone, treat-all, and treat-none strategies.

## 3. Results

A total of 166 patients with rectal cancer were included in the study. The mean age was 63.1 ± 11.5 years (range, 27–84 years), and 60.2% were male. Lymphovascular invasion was observed in 115 patients (69.2%). Histopathology confirmed lymph node metastasis in 122 patients (73.5%), whereas 44 patients (26.5%) were negative for node involvement. The clinical T stages were cT1 in 14 patients (8.4%), cT2 in 48 (28.9%), cT3 in 66 (39.8%), and cT4 in 38 (22.9%) (Table 1).

The distribution of DLD signs differed significantly between the groups (*p* < 0.001). DLD-negative status (Category II) was observed in 83.6% of node-positive patients versus 40.9% of node-negative patients, indicating a strong association between the absence of the DLD sign and nodal metastasis (Table 2).

Node-RADS categories were also significantly associated with nodal status (*p* < 0.001). Lower categories (I–II) were more frequent in node-negative patients, whereas higher categories (III–V) predominated in metastatic cases, particularly Node-RADS III (41.0%), IV (23.8%), and V (15.6%) (Table 2).

The intermediate Node-RADS III category included 59 patients (50 node-positive and 9 node-negative patients), demonstrating substantial heterogeneity. Within the Node-RADS III subgroup, lymph node metastasis was present in 50 patients (84.7%; 95% CI: 73.5–91.8%). DLD-negative cases demonstrated a substantially higher metastasis rate than DLD-positive cases (93.8%, 45/48; 95% CI: 83.2–97.9% vs. 45.5%, 5/11; 95% CI: 21.3–72.0%), suggesting that the DLD sign may provide additional stratification within the indeterminate category.

The combined DLD + Node-RADS model demonstrated improved discrimination for lymph node metastasis compared with either parameter alone. Higher Node-RADS categories combined with DLD-negative status were more frequently associated with metastatic lymph nodes, whereas lower Node-RADS categories combined with DLD-positive status predominated in patients with node-negative status (Table 2). Overall, the combined model provided better separation between node-positive and node-negative patients than either parameter alone.

Inter-reader agreement was moderate for both DLD assessment (κ = 0.56) and the Node-RADS categorization (κ = 0.53).

In the univariate logistic regression analysis, both the Node-RADS score (OR: 2.197; 95% CI: 1.578–3.058; *p* < 0.001) and DLD status (OR: 7.367; 95% CI: 3.415–15.89; *p* < 0.001) were significantly associated with lymph node metastasis (Table 3).

A multivariable logistic regression model, including both the Node-RADS score and DLD status, was constructed. After simultaneous adjustment, both variables remained independently associated with lymph node metastasis. Node-RADS score demonstrated an adjusted OR of 2.054 (95% CI: 1.455–2.899; *p* < 0.001), whereas DLD-negative status remained significantly associated with lymph node metastasis after adjustment for Node-RADS score (*p* < 0.001) (Table 3).

The final multivariable logistic regression equation was as follows:

logit(P) = −4.053 + 0.720 × (Node-RADS score) + 1.892 × (DLD status). The predicted probabilities generated from this equation were subsequently used for the ROC analysis of the combined DLD + Node-RADS model.

Receiver operating characteristic (ROC) analysis based on model-predicted probabilities derived from the multivariable logistic regression model showed that the combined DLD + Node-RADS model achieved the highest diagnostic performance (AUC: 0.795; 95% CI: 0.715–0.875; *p* < 0.001), outperforming both Node-RADS alone (AUC: 0.740) and the DLD sign alone (AUC: 0.713), according to DeLong analysis.

The combined model significantly outperformed both Node-RADS alone (*p* = 0.023) and the DLD sign alone (*p* < 0.001), whereas no significant difference was observed between Node-RADS and the DLD sign alone (*p* = 0.594) (Table 4).

**Figure 4:**
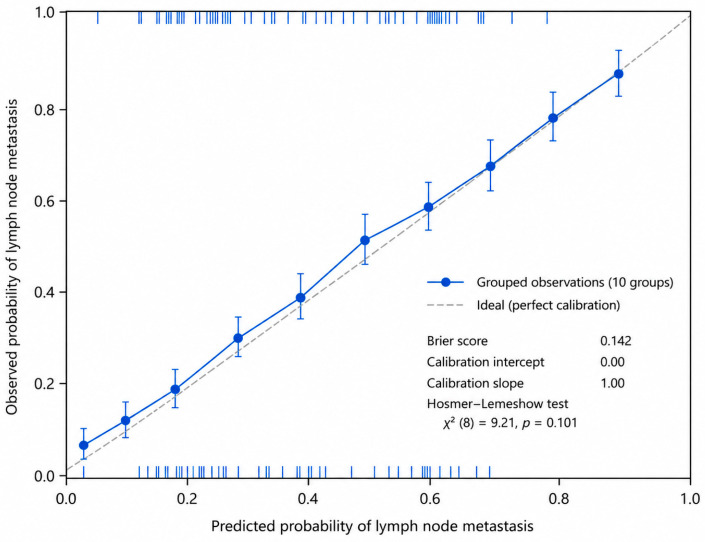
Calibration plot of the combined DLD + Node-RADS model for the prediction of lymph node metastasis in rectal cancer. The dashed diagonal line indicates a perfect agreement between the predicted and observed probabilities. Points represent observed event rates across deciles of predicted risk, and the error bars indicate the 95% confidence intervals.

Calibration analysis demonstrated good agreement between the predicted and observed probabilities. The combined DLD + Node-RADS model achieved a Brier score of 0.142. The Hosmer–Lemeshow goodness-of-fit test showed no evidence of poor calibration (χ^2^ = 9.21, *p* = 0.101). The calibration intercept and slope were approximately 0 and 1, respectively, indicating a good agreement between the predicted and observed outcomes. A calibration plot is shown in Figure 4.

Internal validation using 1000 bootstrap resamples was performed for the combined DLD + Node-RADS model based on the prespecified ROC approach. The apparent AUC was 0.795, the estimated optimism was 0.008, and the optimism-corrected AUC was 0.787, indicating minimal model overfitting and acceptable internal stability.

**Figure 5:**
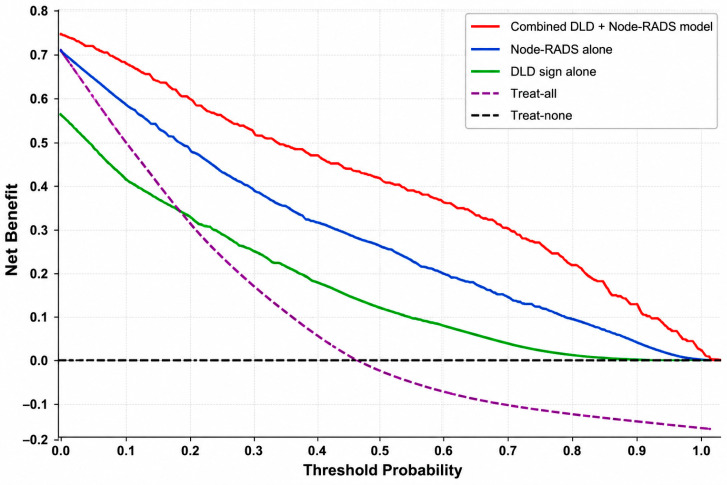
Decision curve analysis comparing the combined DLD + Node-RADS model, Node-RADS alone, DLD sign alone, and default clinical strategies for the prediction of lymph node metastasis. The combined model showed the highest net benefit across a broad range of clinically relevant threshold probabilities.

Decision curve analysis demonstrated that the combined DLD + Node-RADS model provided a higher net benefit than Node-RADS alone, the DLD sign alone, and treat-all and treat-none strategies across a broad range of clinically relevant threshold probabilities, particularly between 0.29 and 0.89. These findings support the potential clinical utility of the combined model beyond statistical discrimination (Figure 5).

## 4. Discussion

In this study, we evaluated the diagnostic performance of the Node-RADS scoring system and the Dark–Light–Dark (DLD) sign for preoperative prediction of lymph node metastasis in rectal cancer and investigated the incremental value of their combined use. Both parameters were significantly associated with nodal metastasis, and the combined model demonstrated a significantly higher discriminatory performance than either method alone, according to the DeLong analysis. These findings suggest that combining a structured morphology-based nodal assessment system with a biologically oriented tumor imaging marker may improve preoperative risk stratification. Because nodal status directly affects neoadjuvant treatment decisions, surgical planning, and prognosis in rectal cancer, an accurate preoperative nodal assessment remains clinically important [14,15,16].

Although MRI is well established for local T staging, accurate N staging remains challenging because of the overlap between benign, reactive, and metastatic lymph nodes. Previous studies have shown that size criteria alone are insufficient, particularly for small metastatic lymph nodes [17,18,19]. In this context, Node-RADS was developed as a structured scoring system that integrates nodal size and morphological characteristics [7]. In our cohort, Node-RADS alone demonstrated moderate diagnostic performance, with an AUC of 0.740, and higher Node-RADS categories were significantly associated with nodal metastasis. This finding is consistent with the findings of prior rectal cancer studies. Niu et al. reported an AUC of 0.826 for the CT-based Node-RADS assessment of mesorectal lymph nodes [20], whereas Jiang et al. demonstrated AUCs ranging from 0.838 to 0.853 across CE-CT, T2WI, and T1CE modalities [21]. A recent meta-analysis by Zhong et al. reported pooled AUC values of 0.92 and 0.91 for Node-RADS ≥ 3 and ≥4 thresholds, respectively, across nine cancer types; however, the overall evidence level was considered weak because of methodological heterogeneity and insufficient positive events [8]. Several factors may explain the lower AUC observed in our study compared to previous reports [8]. First, our cohort consisted predominantly of patients with more advanced disease, as reflected by the high prevalence of nodal metastasis and the exclusion of patients receiving neoadjuvant therapy, resulting in a different case mix than that of prior studies. Second, differences in patient selection, imaging protocols, reader interpretation, and study design may have contributed to the variability in diagnostic performance across studies. Finally, previous investigations often included more heterogeneous populations or multimodal assessment strategies, which may limit the direct comparison of AUC values [20,21].

Because the DLD sign remains a relatively novel imaging biomarker with limited external validation beyond the original study by Yan et al., its clinical role should be considered exploratory [10]. Yan et al. described and validated the DLD sign in a two-center cohort of 1071 patients, demonstrating that its absence on contrast-enhanced T1-weighted imaging was independently associated with lymph node metastasis, with the enhanced model achieving AUCs of 0.89 in the training cohort and 0.83 in the external validation cohort. In our study, DLD-negative status was similarly associated with a markedly higher likelihood of nodal metastasis, supporting the reproducibility of this association in an independent cohort. However, our study differs from that of Yan et al. in two important aspects. First, Yan et al. combined the DLD sign with ESGAR nodal criteria and additional tumor MRI features; however, the present study is the first to evaluate the DLD sign specifically in combination with the Node-RADS scoring system, a structured and site-agnostic nodal assessment framework. Second, our study is the first to investigate the distribution of the DLD sign within the indeterminate Node-RADS category III, which represents a clinically challenging subgroup. The biological basis of the DLD sign has been hypothesized to reflect tumor invasion depth, peritumoral inflammatory response, and muscularis propria integrity. Yan et al. provided histopathologic validation of this trilaminar appearance. However, no dedicated histopathological analysis was performed in this study to investigate these proposed mechanisms. Therefore, the biological interpretation of the DLD sign in our cohort remains inferential and is primarily based on previously published evidence.

Overall, our findings further support the concept that primary tumor imaging characteristics may provide predictive information regarding nodal status beyond conventional nodal morphology alone, although prospective multicenter validation is necessary before clinical implementation.

The principal contribution of the present study is the demonstration that integration of DLD status with the standardized Node-RADS framework provides incremental imaging information beyond morphology-based nodal assessment. The combined model achieved a significantly higher AUC of 0.795 compared to Node-RADS or DLD sign alone, supporting the incremental value of their integration. Importantly, DLD status remained independently associated with lymph node metastasis after adjustment for the Node-RADS score, suggesting that the DLD sign provides complementary information beyond conventional morphology-based nodal assessment. However, this independent association should be interpreted within the context of the present model. Specifically, the reported independent effect reflects adjustment for the Node-RADS score only and does not account for other potentially relevant preoperative clinical or imaging predictors, such as clinical T stage or additional markers of disease severity. Therefore, residual confounding cannot be completely excluded, and further studies incorporating broader preoperative variables are warranted.

The primary objective of the present study was to evaluate the incremental imaging value of the DLD sign beyond Node-RADS, rather than to develop a comprehensive clinicopathological prediction model. Therefore, the multivariate analysis was intentionally restricted to imaging-derived variables. Furthermore, lymphovascular invasion is a postoperative histopathological parameter that is not available at the time of the preoperative MRI assessment.

Notably, the DLD sign appeared particularly informative within the indeterminate Node-RADS III subgroup, in which substantial heterogeneity in nodal status was observed. This finding suggests that the incorporation of a tumor-based imaging marker may further stratify equivocal nodal categories that frequently represent a diagnostic gray zone in clinical practice. Such intermediate-risk cases may substantially influence multidisciplinary treatment decisions, including those regarding neoadjuvant therapy and intensified imaging follow-up. However, this subgroup analysis was exploratory. Therefore, the findings should be interpreted cautiously because of the relatively limited and imbalanced sample size and should be regarded as hypothesis-generating until confirmed in larger independent cohorts.

Recent radiomics- and machine learning–based approaches have shown promising results in predicting lymph node metastasis in rectal cancer [22,23,24]. However, concerns regarding reproducibility, external validity, standardization, and overfitting remain important limitations of many high-dimensional predictive models [25]. In contrast, the DLD sign and Node-RADS can be evaluated using routine MRI sequences without additional software or computational infrastructure. Therefore, the proposed combined approach may represent a more clinically interpretable and practically applicable strategy. Nevertheless, improved discrimination alone does not necessarily translate into meaningful clinical benefits. To further evaluate the clinical usefulness, a decision curve analysis was performed. The combined DLD + Node-RADS model demonstrated a greater net benefit than Node-RADS alone, the DLD sign alone, and default treat-all and treat-none strategies across a broad range of threshold probabilities. These findings suggest that integrating DLD status into the Node-RADS framework may improve clinical decision-making for preoperative nodal risk stratification. Nevertheless, the observed improvement in discriminatory performance was modest (AUC increase from 0.740 to 0.795). Therefore, the present findings should be interpreted as demonstrating the potential incremental value of integrating DLD status with Node-RADS rather than providing sufficient evidence to support routine clinical adoption. Prospective multicenter studies with external validation are required before implementation in routine clinical practice can be recommended. Further studies incorporating formal reclassification metrics, such as NRI and IDI, may help better quantify the incremental value of the combined model. Because no single probability threshold was prespecified for clinical decision-making, threshold-dependent operating characteristics (sensitivity, specificity, positive predictive value, and negative predictive value) were not reported to avoid post hoc selection of an optimal cutoff.

Predictive models should also be evaluated using calibration and decision curve analyses and external validation in independent cohorts. In the present study, calibration analysis demonstrated good agreement between the predicted and observed outcomes, with a Brier score of 0.142 and a non-significant Hosmer–Lemeshow test result. These findings suggest that the combined model was reasonably well-calibrated within the study cohort. Furthermore, bootstrap internal validation supported the internal stability of the combined model; however, external validation in independent cohorts remains necessary before clinical implementation, and the reported diagnostic performance should be considered preliminary and may be subject to some degree of optimism inherent to model development within a single retrospective cohort. It should be emphasized that these calibration results represent apparent model performance within the development cohort and, despite bootstrap internal validation, should not be interpreted as evidence of external calibration. Confirmation of calibration performance in independent external cohorts is required before routine clinical implementation can be recommended.

This study has several limitations. The most important limitation of this study is its retrospective single-center design combined with the high prevalence of lymph node metastasis in the study cohort (73.5%). The relatively high prevalence of lymph node metastasis (73.5%) in our cohort likely reflects the specific characteristics of the study population. Patients who received neoadjuvant therapy before MRI were excluded to preserve direct radiologic-pathologic correlation, resulting in an enrichment of surgically treated patients with biologically more advanced disease. In addition, our institution functions as a tertiary referral center, which may further increase the proportion of patients with high-risk tumors. Consequently, the study cohort may not fully represent the broader spectrum of rectal cancer encountered in routine clinical practice, particularly early stage disease, and caution is warranted when extrapolating the reported predictive values to populations with a lower nodal metastasis prevalence.

In addition, patients who underwent neoadjuvant therapy before imaging were excluded to preserve pathologic correlation and cohort homogeneity; consequently, the study population may not fully represent the broader oncologic population encountered in routine clinical practice. Although calibration, bootstrap internal validation, and decision curve analyses were performed, external validation and formal net reclassification analyses were not performed. Therefore, the reproducibility, generalizability, and clinical utility of the model must be confirmed in prospective multicenter cohorts before routine clinical implementation.

Although the combined model demonstrated significantly improved discriminatory performance according to the DeLong analysis, confirmation of reproducibility and clinical utility in prospective multicenter cohorts remains necessary before routine clinical implementation. Furthermore, although the inter-reader agreement was moderate, the interpretation of the DLD sign may still be reader-dependent. Nevertheless, a blinded independent assessment and consensus review were conducted to improve interpretive consistency. Individual reader diagnostic performance was not separately evaluated because the primary analysis was intentionally based on consensus interpretations. Consequently, the incremental benefit of consensus assessment over individual reader performance could not be formally quantified. Furthermore, pairwise inter-reader agreement analyses and reader-specific diagnostic performance before consensus adjudication were not assessed; therefore, the influence of reader experience on diagnostic performance could not be formally evaluated. As a result, the reproducibility of DLD assessment should be considered preliminary. Although the observed inter-reader agreement was acceptable for an exploratory imaging biomarker study (κ = 0.53–0.56), it remained only moderate and may limit immediate clinical implementation. Accordingly, additional multicenter studies incorporating formal reproducibility analyses and readers with different levels of experience are warranted before widespread clinical adoption can be recommended.

In addition, the analysis was performed on a patient basis rather than a node-by-node basis. Individual lymph node counts and node-specific radiologic–pathologic matching were not available because of the retrospective study design. Therefore, node-to-node pathological correlations could not be performed. Furthermore, because the highest Node-RADS score was selected to represent each patient, some degree of aggregation bias could not be excluded. Consequently, the lymph nodes analyzed on MRI may not necessarily correspond to the specific metastatic lymph nodes identified on histopathology.

Finally, no dedicated histopathological correlation was performed to investigate the proposed biological basis of the DLD sign. Therefore, mechanistic interpretations of tumor invasion depth, inflammatory response, and muscularis propria integrity could not be confirmed in the present cohort. In addition, potentially relevant clinicopathological variables, such as the clinical T stage, were not incorporated into the multivariable model. Therefore, residual confounding related to the overall disease severity cannot be completely excluded.

Future multicenter studies incorporating external validation cohorts, node-specific radiologic–pathologic correlation, and integration with advanced quantitative imaging biomarkers may further clarify the clinical role of the combined DLD + Node-RADS approach and support its implementation in routine rectal cancer staging.

## 5. Conclusions

The integration of the DLD sign with the Node-RADS scoring system significantly improved the preoperative prediction of lymph node metastasis in rectal cancer compared with either approach alone. These findings suggest that integrating morphology-based nodal assessment with biologically oriented tumor imaging features may improve preoperative nodal risk stratification, particularly in indeterminate categories such as Node-RADS III. Nevertheless, external validation is required before routine clinical implementation.

## Figures and Tables

**Table 1:** Baseline demographic, clinical, and imaging characteristics of the study population.

Variable	Min–Max	Median	Mean ± SD/n (%)
**Age (years)**	27–84	64	63.1 ± 11.5
**Age Group**			
<65 years			84 (50.6%)
≥65 years			82 (49.4%)
**Sex**			
Male			100 (60.2%)
Female			66 (39.8%)
**Dark–Light–Dark (DLD) Sign**			
Score I (Positive)			46 (27.7%)
Score II (Negative)			120 (72.3%)
**Node-RADS Score**			
I			30 (18.1%)
II			22 (13.3%)
III			59 (35.5%)
IV			33 (19.9%)
V			22 (13.3%)
**Lymphovascular Invasion**			
Negative			51 (30.8%)
Positive			115 (69.2%)
**Lymph Node Involvement**			
Negative			44 (26.5%)
Positive			122 (73.5%)
**Clinical T stage, n (%)**			
1			14 (8.4%)
2			48 (28.9%)
3			66 (39.8%)
4			38 (22.9%)

**Table 2:** Association between imaging parameters and histopathologically confirmed lymph node metastasis.

		Lymph Node Negative (n = 44)		Lymph Node Positive (n = 122)		*p*	
		n	%	n	%		
**Dark–Light–Dark (DLD) Sign**	I	26	59.1%	20	16.4%	*p* < 0.001	χ^2^
	II	18	40.9%	102	83.6%		
**Node-RADS Score**	I	19	43.2%	11	9.0%	*p* < 0.001	χ^2^
	II	9	20.5%	13	10.7%		
	III	9	20.5%	50	41.0%		
	IV	4	9.1%	29	23.8%		
	V	3	6.8%	19	15.6%		
χ^2^ = Chi-square test							

**Table 3:** Univariate and multivariable logistic regression analyses of Node-RADS score and DLD status for the prediction of lymph node metastasis.

Variable	Univariate Model			Multivariate Model		
	OR	95% CI	*p* Value	OR	95% CI	*p* Value
**Node-RADS Score**	2.197	1.578–3.058	<0.001	2.054	1.455–2.899	<0.001
**Dark–Light–Dark (DLD) Sign**	7.367	3.415–15.89	<0.001	6.635	2.873–15.319	<0.001

**Table 4:** Receiver operating characteristic curve analysis of the imaging parameters.

Parameter	Area Under the Curve (AUC)	95% Confidence Interval	*p* Value
**Combined DLD + Node-RADS Model**	0.795	0.715–0.875	<0.001
**Node-RADS Score**	0.740	0.648–0.832	<0.001
**Dark–Light–Dark (DLD) Sign**	0.713	0.618–0.809	<0.001

## Data Availability

No new data were created or analyzed in this study. Data sharing is not applicable to this article.
